# A single-mode data acquisition architecture for PET/MRI

**DOI:** 10.1186/2197-7364-2-S1-A19

**Published:** 2015-05-18

**Authors:** Giancarlo Sportelli, Nicola Belcari, Maria Giuseppina Bisogni, Niccolo Camarlinghi, Emanuele Zaccaro, Alberto Del Guerra

**Affiliations:** Department of Physics, University of Pisa and INFN, Pisa, Italy

The development of MRI compatible detectors based on compact solid state photomultipliers has recently led to simultaneous fully integrated PET/MRI systems for human imaging. The PET acquisition design for MRI integration is known to have several additional constraints, including smaller space, electromagnetic compatibility issues and thermal management. The current work presents the PET acquisition architecture that has been developed for the TRIMAGE project, whose aim is to provide a cost effective, commercial grade trimodality PET/MRI/EEG scanner. The TRIMAGE PET component consists of 216 modules of 2.5 cm x 2.5 cm, arranged in 18 rectangular detectors of 5 cm x 15 cm, the latter in the axial direction, to form a full ring of 31 cm diameter. Each module consists of a staggered dual layer LYSO matrix read out by two arrays of 4 x 8 SiPMs and an ASIC. The detector board hosts a low-power low-end FPGA that performs pixel identification, energy calibration and handles the communication between the ASICs and the motherboard, which is located in proximity of the scanner. Data is streamed using high-density shielded cables and high-speed LVDS transmission to 9 low-end SoC FPGAs and from there to a central mainboard where coincidences and events statistics are processed. Coincidence data is finally transmitted to a host PC for image reconstruction. The proposed architecture and technological solutions will be presented and discussed.

